# Cloning and Expression of the Organophosphate Pesticide-Degrading *α*-*β* Hydrolase Gene in Plasmid pMK-07 to Confer Cross-Resistance to Antibiotics

**DOI:** 10.1155/2018/1535209

**Published:** 2018-05-16

**Authors:** Kirubakaran Rangasamy, Murugan Athiappan, Natarajan Devarajan, Javid A. Parray, Nowsheen Shameem, K. N. Aruljothi, Abeer Hashem, Abdulaziz A. Alqarawi, Elsayed Fathi Abd_Allah

**Affiliations:** ^1^Department of Microbiology, Periyar University, Salem, Tamil Nadu, India; ^2^Department of Biotechnology, Periyar University, Salem, Tamil Nadu, India; ^3^Department of Environmental Science, Government SAM Degree College Budgam, Jammu & Kashmir 191111, India; ^4^Department of Environmental Science, Cluster University Srinagar, Jammu & Kashmir 190001, India; ^5^Department of Genetic Engineering, SRM University, Chennai, Tamil Nadu, India; ^6^Botany and Microbiology Department, College of Science, King Saud University, P.O. Box 2460, Riyadh 11451, Saudi Arabia; ^7^Mycology and Plant Disease Survey Department, Plant Pathology Research Institute, Agriculture Research Center, Giza, Egypt; ^8^Plant Production Department, College of Food and Agricultural Sciences, King Saud University, P.O. Box 2460, Riyadh 11451, Saudi Arabia

## Abstract

Pesticide residual persistence in agriculture soil selectively increases the pesticide-degrading population and transfers the pesticide-degrading gene to other populations, leading to cross-resistance to a wide range of antibiotics. The enzymes that degrade pesticides can also catabolize the antibiotics by inducing changes in the gene or protein structure through induced mutations. The present work focuses on the pesticide-degrading bacteria isolated from an agricultural field that develop cross-resistance to antibiotics. This cross-resistance is developed through catabolic gene clusters present in an extrachromosomal plasmid. A larger plasmid (236.7 Kbp) isolated from* Bacillus* sp. was sequenced by next-generation sequencing, and important features such as *α*-*β* hydrolase, DNA topoisomerase, DNA polymerase III subunit beta, reverse transcriptase, plasmid replication rep X, recombination U, transposase, and S-formylglutathione hydrolase were found in this plasmid. Among these, the *α*-*β* hydrolase enzyme is known for the degradation of organophosphate pesticides. The cloning and expression of the *α*-*β* hydrolase gene imply nonspecific cleavage of antibiotics through a cross-resistance phenomenon in the host. The docking of *α*-*β* hydrolase with a spectrum of antibiotics showed a high G-score against chloramphenicol (−3.793), streptomycin (−2.865), cefotaxime (−5.885), ampicillin (−4.316), and tetracycline (−3.972). This study concludes that continuous exposure to pesticide residues may lead to the emergence of multidrug-resistant strains among the wild microbial flora.

## 1. Introduction

Plasmid-borne drug resistance and its persistence among soil bacteria cause great public health hazards. Although other genetic elements such as nonconjugative, mobilizable plasmids contribute to multidrug resistance [1–3], bacterial plasmid DNA also confers drug resistance [4, 5]. Multidrug resistance is very common among soil bacteria that are exposed to pesticides [6, 7]. Moreover, the indiscriminate use of pesticides favours the microbial population that can metabolize those pesticides. Higher exposure to pesticides enables bacteria to produce suitable enzymes for the degradation of pollutants. Consequently, pesticide-metabolizing populations tend to overgrow [8–10]. Pesticide-degrading bacteria metabolize the pesticides through hydrolytic cleavage for their carbon and energy sources [11, 12]. Degradation of pesticides may also occur through oxidation, reduction, hydrolysis, peroxidise, or oxygenase mechanisms [13, 14].

Enzymes produced by pesticide-degrading bacteria can catabolize drugs and other xenobiotics. Breaking C=C bonds in the drug by nonspecific cleavage leads to cross-resistance to antibiotics [15, 16]. This could be an explanation regarding how susceptible soil bacteria become multidrug resistant. A previous study on formaldehyde resistance with* Enterobacteriaceae* has shown high formaldehyde dehydrogenase activity leading to multidrug resistance through nonspecific activity [17, 18]. A study conducted on formaldehyde dehydrogenase enzyme-mediated multidrug resistance provided the notion that cross-resistance is possible via nonspecific degradation. Another study proved that enzymes produced by soil bacteria in pesticide-contaminated soil can degrade other xenobiotics [19–21]. Assessing the catabolic properties of *α*-*β* hydrolase, an enzyme-degrading pesticide, would answer whether these enzymes play a role in multidrug resistance. It is very common among soil bacteria to adapt themselves to changing environmental conditions. Evolutionarily, bacteria in a stressed environment tend to produce putative enzymes via induced mutations that can degrade a wide range of xenobiotics [22].

Therefore, the present study aimed to understand the bacterial isolates with an extrachromosomal plasmid carrying pesticide-resistant genes that also confer the cross-resistance of multidrug resistance. In silico analyses were performed to understand the mechanism of cross-resistance via the *α*-*β* hydrolase enzyme.

## 2. Materials and Methods

### 2.1. Sample Collection

Soil samples were collected from up to 5 cm of the upper layer from different pesticides applied to an agriculture field located in the Salem district (11.7794°N, 78.2034°E), Tamil Nadu, India. The collected samples were transported to the laboratory and stored at 4°C until further analysis.

### 2.2. Plasmid Isolation and DNA Sequencing

Owing to the increased use of the plasmid, the sample was kept for plasmid enrichment by incubating with 1% pesticide (monocrotophos) supplemented medium for two days. The bacterial strains were isolated from the enriched sample, and it was found that* Bacillus* sp. MK-07 (KU510395.1) was predominant. Plasmid DNA (pMK-07) was isolated by the alkaline lysis method from the isolate [23]. The plasmid was treated with plasmid-safe, adenosine 5-triphosphate- (ATP-) dependent DNase to remove any genomic DNA contamination. The plasmid DNA was processed for library preparation using an Illumina Nextera XT DNA library preparation kit. SnapGene version 4.0.2 software was used to create a plasmid DNA map [24]. The library was sequenced on MiSeq using 2 × 300 bp to generate approximately 1 GB of data. The Draft assemblies of short Illumina sequence reads (2 × 300 MiSeq library) were analysed with a 4200-tape station system, Eurofins Genomics, Bangalore, India (Agilent Technologies, USA) [25].

### 2.3. Assembling the Plasmid DNA Sequence

Raw data were processed using Trimmomatic v 0.35 to remove adapter sequences, ambiguous reads (reads with unknown nucleotides “N” larger than 5%), and low-quality sequences (reads with more than 10% quality thresholds (QV) < 20 phred score). Clear sequences with a size of 1,073,566 (2 × 300 bp) high-quality reads were retained for further analysis and were used for de novo assembly [2, 26].

#### 2.3.1. Gene Prediction and Functional Annotation

Sequences were predicted using prodigal with default parameters. In total, 225 genes were predicted with an average gene size of 816 bp, while the maximum and minimum sizes of the genes were 15,033 bp and 105 bp, respectively [27, 28]. Gene ontology annotations of the predicted [29] genes were determined by the Blast2GO program (https://www.Blast2GO.com). Gene ontology assignments were used to classify the functions of the predicted genes. Functional annotations of the genes were performed using BLASTx, part of the NCBI-Blast-2.3.0 standalone tool. BLASTx was used to find the homologous sequence of genes against NR (nonredundant protein database) within* Bacillus cereus* (MK-07).

### 2.4. Phylogenetic Distinct Clades and Cloning of *α*-*β* Hydrolase Gene

The scaffold sample of plasmid (pMK-07) was aligned against plasmids of all the* Bacillus* species using BlastN. A Newick file was downloaded from the Blast Tree View and plotted further using an interactive tree of life (http://itol.embl.de/upload.cgi). Different parameters were adjusted according to the visualization requirements and were exported [29]. The *α*-*β* hydrolase gene was amplified using a gene-specific primer that was designed by the net primer (Premier Biosoft): *α*-*β* hydrolase MK-FP: ATGGCTAAAGAAATGTTTGTGC and MK-RP: CGCACTAACTACTACTTCTGGT. The polymerase chain reaction mixtures (50 *μ*l) contained 10 *μ*M of each primer, PCR Invitrogen Master Mix (PCR buffer, 5 U of Taq polymerase, 10 *μ*M of BSA and 2 *μ*l of DNA). The thermocycling conditions included a denaturation step at 94°C for 3 min, 34 amplification cycles of 94°C for 1 min, 57°C for 30 sec and 72°C for 1 min, and a final extension step for 8 min using an Eppendorf thermocycler (Eppendorf AG 22331). Electrophoresis was continued for 30 min at 100 V (Tarson electrophoresis unit). The size of the fragment was determined by comparing it with a 1 kb marker (NEB). The gene product was inserted into the pXcm vector using a ligation (Fermentas) enzyme.

The *α*-*β* hydrolase gene was released from the pXcm vector using Bam H1 and Hind III. Expression of *α*-*β* hydrolase in* E. coli *DH5*α* was achieved by subcloning it into pET-20. Transformation of recombinant DNA into* E.coli/DH5α*: pET-20b was carried out by standard methods [30]. Preliminary screening was performed based on the blue-white colonies on x-gal medium, followed by PCR amplification of the *α*-*β* hydrolase gene.

The recombinant bacterial strains were cultured overnight. The cells were harvested by centrifugation, and *α*-*β* hydrolase was recovered by sonication (10–15 min). Crude enzyme was electrophoresed by slope gel electrophoresis along with marker protein (SERVA) and then analysed. Next, 100 *μ*l of crude enzyme was mixed with 1 ml of 30 *μ*g/ml chloramphenicol, followed by incubation at 37°C for 48 hours. After the incubation, the metabolites were purified with twofold ethyl acetate and were evaporated under vacuum conditions. The extracted residues were dissolved in methanol to a volume of 2 ml and were stored at 4°C until GC-FID analysis. The extract was analysed in an Agilent gas chromatograph (Model 7820A Series USA) equipped with a flame ionization detector [31].

### 2.5. Docking with Ligands

The Crystal Structure of *α*-*β* hydrolase (PDB ID: 1I6W) was retrieved from the protein data bank, and the ligands were downloaded from PubChem (http://www.ncbi.nlm.nih.gov/pccompound) with a PubChem ID (Table 1). The ligands were retrieved from the PubChem (http://www.ncbi.nlm.nih.gov/pccompound) database based on a literature survey. These compounds were subjected to ligand preparation by the Ligprep wizard application of the Maestro 9.2. Corrections such as the addition of hydrogen, 2D to 3D conversion, corrected bond lengths and bond angles, low energy structure, stereochemistries, and ring conformation, followed by minimization and optimization in the optimized potential for the liquid simulation force field [32–34] were performed. One conformation for each ligand was generated with other parameters used as the default in Maestro 9.2. Protein-ligand binding sites were predicted by the core-attachment based method (COACH) (http://zhanglab.ccmb.med.umich.edu/COACH/) using the meta-server approach. Complementary ligand binding site predictions were achieved using two comparative methods, TM-SITE and S-SITE, which recognize ligand binding templates from the BioLiP protein function database by binding-specific substructure and sequence profile comparisons. Docking was performed using the Glide software package (http://www.schrodinger.com/), which searches for favourable interactions between one or more typically small ligand molecules and a larger receptor molecule, usually a protein. The retrieved structures were subjected to the removal of water up to 5-Å distances, assigning lone pair electron atoms using a protein preparation wizard. The receptor grid was set up and generated to specify the binding pocket where the ligand binds using the receptor grid generation panel. Molecular docking of the prepared protein and ligand was carried out using Glide.

## 3. Results and Discussion

Continuous usage and accumulation of pesticide in the agricultural field lead to the development of cross-resistance to antibiotics among soil bacteria. Plasmid DNA (pMK-07) from* Bacillus *sp. was sequenced and analysed using in silico tools, revealing that the plasmid DNA sequences and their relatedness lead to cross-resistance to pesticide and antibiotics. A triclosan-resistant bacterial population showing resistance to antimicrobial agents [35, 36] due to self-transmissible genes that can jump between plasmids and chromosomes [37, 38] and the accumulation of multidrug resistance genes in the soil bacterial community through horizontal gene transfer were common among pesticide degraders [39, 40]. Thus, these studies proved the phenomenon of cross-resistance in bacteria.

### 3.1. Plasmid DNA Sequence

**Figure 1:**
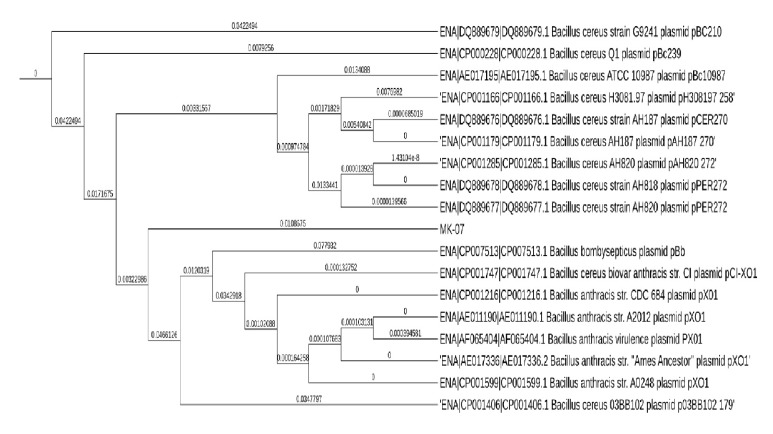
Homology cladogram of the plasmid DNA (sequence similarity of pMK-07 with* Bacillus cereus*).

The sequencing of plasmid pMK-07 of* Bacillus* species isolated from pesticide-exposed agriculture soil revealed that the plasmid shares genes from 6 different strains of* Bacillus cereus *(MSX-A12, NC7401, AH187, MSX-D12, IS845/00, and H3081.97),* B. weihenstephanensis, *and* S. pneumoniae. *Phylogenetic and dendrogram analyses of pMK-07 revealed that the plasmid shares 100% sequence similarity with* Bacillus* species (Figure 1), and the sequence was deposited in GenBank (KY940428.1).

In total, 225 genes were annotated from the plasmid, among which 221 genes found hits in the nucleotide database and four genes did not have a matching sequence in the database. Based on gene ontology annotation, the genes from the plasmid were categorized into three domains: biological process, cellular component, and molecular function. Similar categorization was performed for a plasmid (pNUC and p11601MD) that includes cellular and molecular component genes of a clinical multidrug resistance in* S. typhimurium* and* Campylobacter jejuni* strain 11601MD [2, 3] (Table 2).

### 3.2. Biological and Molecular Function of Genes

The genes responsible for the biological process of the bacteria, including the genes for spore formation, germination, sporulation-specific N-acetylmuramoyl-L-alanine amidase, small, acid-soluble spore protein C_5_, and germination protein-*Ger *(x) C family protein, were found. These genes enable the bacteria to withstand the adverse conditions. The genes that are essential for DNA recombination were also present in the plasmid: site-specific recombinase, resolvase family, Tn1546 resolvase recombination protein U, and integrase* (Bacillus cereus)* [4]. Thus, the plasmid underwent random recombination with different strains of* Bacillus *sp. The presence of genes such as thetraG/traD family, Flp pilus assembly protein, and* cpa*B determines the horizontal gene transfer through conjugation [41, 42]. The presence of the ars R regulatory element makes the bacteria sense the presence of metal ions in the surroundings [43] and develop tolerance against the metal ions. The gene* rep X *present in the plasmid is known for plasmid DNA replication. The presence of the IS3 and IS605 transposase families allows the DNA-mediated recombination and insertion of random sequences in the bacterial genome and extrachromosomal plasmid DNA. The presence of RNA-mediated DNA polymerase (reverse transcriptase) genes indicated a history of involvement of viral transduction in the route of de novo plasmid generation.

**Figure 2:**
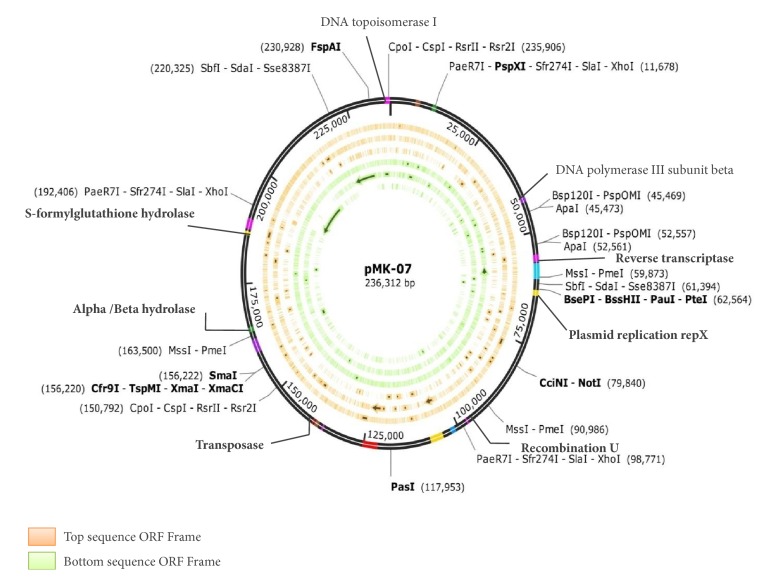
Gene map of plasmid DNA (pMK07) (distribution of *α*-*β* hydrolase, DNA topoisomerase, DNA polymerase III subunit beta, reverse transcriptase, plasmid replication rep X, recombination U, transposase, and S-formylglutathione hydrolase).

DNA sequence analysis of the plasmid (pMK-07) DNA revealed the genes harboured in the novel de novo plasmid pMK-07 (Figure 2). Previous studies have shown that *α*-*β* hydrolase can hydrolyse a wide range of pesticides [44], and glutathione S-transferases (GSTs) were found to hydrolyse DDT [45, 46], organochlorine, and organophosphorus insecticides [47, 48]. Hydrolases and hypothetical protein existence in the plasmid suggest that it was a degradative plasmid, especially pesticides [49].

### 3.3. Cellular Components

It was observed that approximately 10% of genes are present in the plasmid codes for membrane components. The bacteria possess an LPXTG anchoring domain and sortase enzyme genes, whose coexistence affirms that the bacteria carrying this pMK-07 plasmid might also be pathogenic [50].

### 3.4. Evolution of Newer Characters

The increased uses of pesticides in the agricultural field serve as the selection pressure for the evolution of soil microbial flora. The bacteria in the soil tend to develop tolerance by acquiring new genes or plasmids from other bacterial sources by either vertical or horizontal gene transfer. Surprisingly, plasmids carry all the essential genes required for survival under adverse or stressed conditions, a finding that has been confirmed in* C. jejuni* and* E. coli* [51]. A similar observation was noted among plant pathogenic Gram-negative bacteria carrying genes essential for their infection in plants [52]. The results from our study agree with those in previous studies.

### 3.5. Cloning of the *α*-*β* Hydrolase Gene

**Figure 3:**
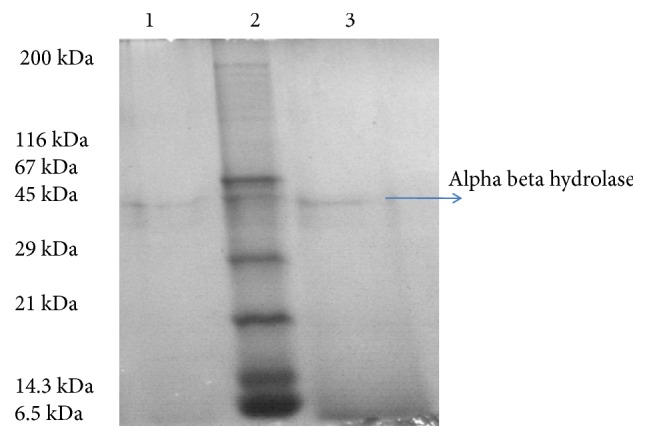
Characterization of *α*-*β* hydrolase SDS-PAGE gels (lane-1: XL1 blue MRF':pET *α*-*β* hydrolase gene expression; lane-2: SERVA unstained SDS-PAGE protein marker; lane-3:* Bacillus *sp. expression enzyme).

The *α*-*β* hydrolase gene (700 bp) was cloned into pXcm and was confirmed for their presence by running it on a 1.0% agarose gel. The expression of the gene for the *α*-*β* hydrolase enzyme was also verified by SDS-PAGE, with the protein size corresponding to 45 kDa (Figure 3). The *α*-*β* hydrolase gene from the pXcm vector was then excised and subcloned into pET-20b. After transformation, the bacterial cells were screened on LB agar medium supplemented with ampicillin, IPTG, and X-gal. Plates showing white colonies (transformants; pET-*α*-*β* hydrolase plasmid) were picked and processed for further use.

### 3.6. Nonspecific Degradation of Chloramphenicol

**Figure 4:**
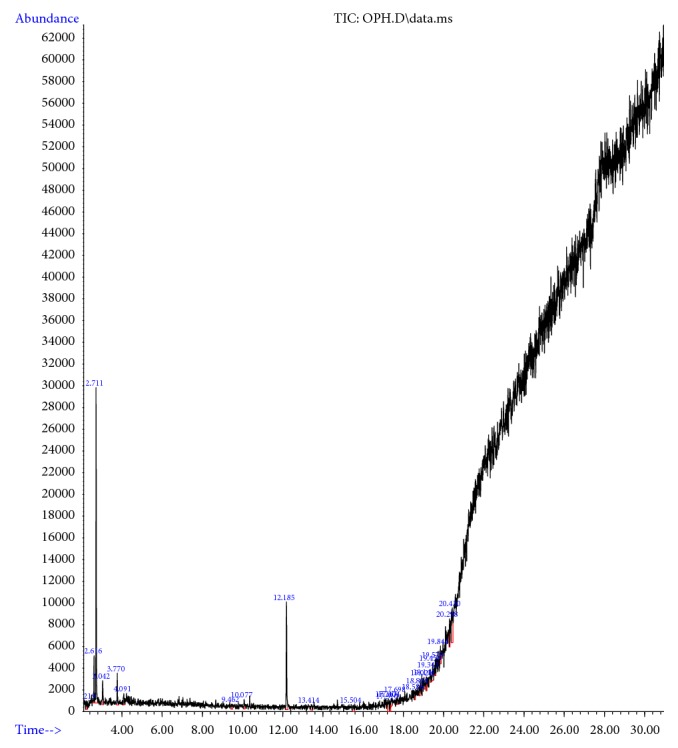
Nonspecific degradation of chloramphenicol by *α*-*β* hydrolase analysed by GC-MS (nonspecific degradation of antibiotics with *α*-*β* hydrolase analysed by GC-MS).

**Figure 5:**
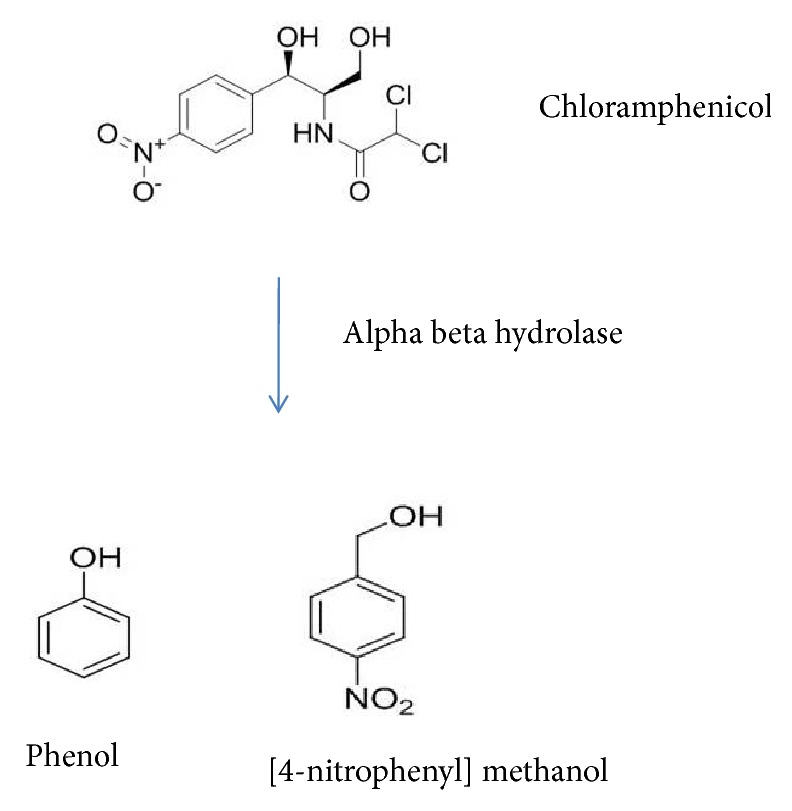
Pathway depicting chloramphenicol metabolism by *α*-*β* hydrolase (nonspecific degradation of chloramphenicol produces 4-nitrophenyl methanol and phenol as intermediates).

It was predicted that *α*-*β* hydrolase could degrade chloramphenicol by nonspecific cleavage and break the C=C bond in the ring structure. Similar phenomena were observed in our study, when the cell lysate was mixed with 30 *μ*g/ml of chloramphenicol, the lysate degraded the antibiotic, an observation that was proven through GC-MS analysis (Figure 4). GC-MS analysis revealed the breakdown compounds present in the pET *α*-*β* hydrolase-treated sample (methane, oxybis dichloro, phenol, indole-2-one) (Figure 5). Based on previous works on the characterization of the catabolic ability of *α*-*β* hydrolase, the side chain of the nucleophilic amino acid residue of the enzyme attacks the electropositive carbon atom of the substrate [53–55]. The findings of the present study suggest that this bacterial strain,* Bacillus *sp. MK-07, which survived all the sublethal concentration of pesticides, potentially has the cross-resistance property to degrade the antibiotic chloramphenicol. The cross-resistance mechanisms may be due to ribosomal gene alteration to evolve cross-resistance [7].

### 3.7. Protein-Ligand Binding Site Prediction

The *α*-*β* hydrolase ligand binding sites were predicted by COACH. The number of templates as the Cluster size was 69, the confidence score (c-score) was 0.96, and the binding residues were VAL9, HIS 10, VAL 74, ALA 75, HIS 76, ASP 103, ASP 133, and VAL 154 (Table 3).

#### 3.7.1. Protein-Ligand Interaction

*α*-*β* hydrolase (PDB Id: 1I6W) was docked with antibiotics (chloramphenicol, streptomycin, cefotaxime, ampicillin, and tetracycline) and the pesticide monocrotophos using Glide Maestro 9.2. Identification of the best-fit antibiotic was performed based on the G-score and number of hydrogen bonds involved. A similar study showed that a sublethal concentration of herbicides would result in the development of multidrug resistance among soil bacteria [56]. Because of the toxicity of the pesticide, the bacteria develop resistance, which allows them to adapt to such components [57].

**Figure 6:**
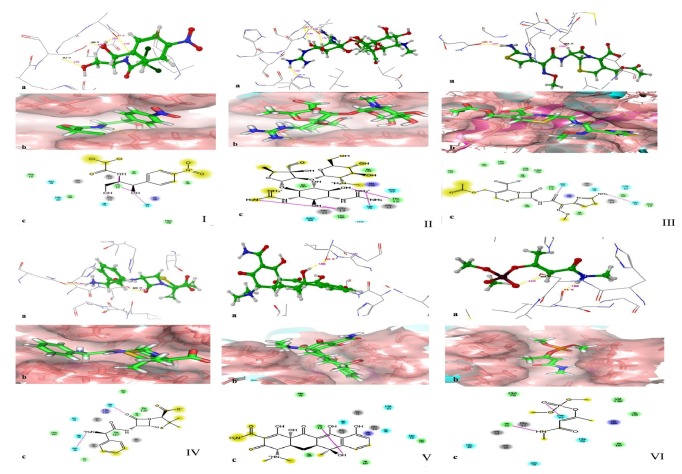
Docking of *α*-*β* hydrolase with antibiotics. Interaction of GLY 11 and SER 77 with the OH group. Similarly, ILE 12 binds to NH and OH; II: interaction of ILE 12 with the OH group. HIE 76 binds to NH, and GLY 13 binds to both the NH and OH groups; III: binding interaction of ASN 18 with the OH group and SER 77 with the NH group; IV: interaction of ASN 18 with the NH_3_ group. SER 77 binds to the OH group; V: docking interaction of ILE 12 binding to the OH group; VI: docking interaction of ILE 12 binding to the NH group. SER 77 interacts with the OH group.

The strong interaction of *α*-*β* hydrolase with chloramphenicol showed a Glide score of −3.793 Kcal/mol. Chloramphenicol interacts with ILE 12, GLY 11, and SER 77 with distances of 2.214 Å and 2.176 Å, 2.255 Å, and 2.430 Å, respectively, at the active site of the enzyme (Figure 6. I.a). In the surface view of the *α*-*β* hydrolase-chloramphenicol complex, chloramphenicol is highlighted with green (Figure 6. I.b). In the 2D interaction of the *α*-*β* hydrolase-chloramphenicol complex, the purple dotted line represents the hydrogen bond with the side chain (Figure 6. I.c and Table 3).

The surface view of *α*-*β* hydrolase with streptomycin showed a Glide score of −2.865 Kcal/mol and interaction of streptomycin with ILE 12, GLY 13, and HIS 76 at the active site (Figure 6. II.a). In the surface view of the *α*-*β* hydrolase-streptomycin complex, streptomycin is highlighted in green (Figure 6. II.b). In the 2D interaction of the *α*-*β* hydrolase-streptomycin complex, the purple dotted line represents the hydrogen bond with the side chain (Figure 6. II.c and Table 3).

The interaction formed between *α*-*β* hydrolase and cefotaxime showed a Glide score of −5.885 Kcal/mol. This interaction of cefotaxime at the active site of the enzyme with ASN 18 and with SER 77 revealed distances of 2.077 Å and 2.022 Å, respectively (Figure 6. III.a). In the surface view of the interaction of the *α*-*β* hydrolase-cefotaxime complex, cefotaxime is highlighted in green (Figure 6. III.b). In the 2D interaction of the *α*-*β* hydrolase-cefotaxime complex, the purple dotted line represents the hydrogen bond with the side chain (Figure 6. III.c and Table 3).

The interaction formed between *α*-*β* hydrolase and ampicillin showed a Glide score of −4.316 Kcal/mol. This interaction involves two hydroxyl bonds between the hydrogen atom of ampicillin with ASN 18 and an oxygen atom of ampicillin with SER 77 with a distance of 1.987 Å and 2.181 Å (Figure 6. IV.a). In the surface view of the *α*-*β* hydrolase-ampicillin complex, ampicillin is highlighted in green (Figure 6. IV.b). In the 2D interaction of the *α*-*β* hydrolase-ampicillin complex, the purple dotted line represents the hydrogen bond with the side chain (Figure 6. IV.c and Table 3).

The interaction formed between *α*-*β* hydrolase and tetracycline showed a Glide score of −3.972 Kcal/mol. This interaction involved ILE 12 hydroxyl bonds between tetracycline and ILE 12 with a distance of 1.826 Å (Figure 6. V.a). In the surface view of the *α*-*β* hydrolase-tetracycline complex, tetracycline is highlighted in green (Figure 6. V.b). In the 2D interaction of the *α*-*β* hydrolase-tetracycline complex, the purple dotted line represents the hydrogen bond with the side chain (Figure 6. V.c and Table 3).

The interaction of *α*-*β* hydrolase with monocrotophos showed a Glide score of −4.464 Kcal/mol. The monocrotophos interacts with ILE 12 and with SER 77 with a distance of 1.865 Å and 2.210 Å at the active site of the enzyme (Figure 6. VI.a). In the surface view of the *α*-*β* hydrolase-monocrotophos complex, monocrotophos is highlighted in green (Figure 6. VI.b). In the 2D interaction of the *α*-*β* hydrolase-monocrotophos complex, the pink dotted line represents the hydrogen bond with the side chain (Figure 6 VI.c and Table 3). Among these antibiotics, based on the docking scores, it can be concluded that all five antibiotics can be degraded through nonspecific cleavage by *α*-*β* hydrolase.

A similar observation in the present study has proven that the hydrolase enzymes could bind with chloramphenicol and hydrolyse it into a nontoxic substance [58, 59]. Excessive pesticide usage resulted in the accumulation of pesticide residues in crops, soils, and the biosphere, creating ecological stress [60, 61].

## 4. Conclusion

The present work focuses on the pesticide-degrading bacteria isolated from an agricultural field that develop cross-resistance to antibiotics. This cross-resistance is developed through catabolic gene clusters present in an extra chromosomal plasmid. It can be concluded from the current study that existence of pesticide-resistant plasmids among soil bacteria can also confer cross-resistance to antibiotics through natural selection exerted by pesticide accumulation in the agriculture field. The enzymes that degrade pesticides can also catabolize the antibiotics by inducing changes in the gene or protein structure through induced mutations. Hence, an alternate way to control pests may pave the way for limiting the emergence of multidrug resistance.

## Figures and Tables

**Table 1:** List of ligands used for the docking analysis with *α*-*β* hydrolase.

Compounds	Molecular Weight	Pubchem ID
Chloramphenicol	323.132	5959
Streptomycin	581.574	19649
Cefotaxime	455.47	6540461
Ampicillin	349.41	6249
Tetracycline	444.435	54675776
Monocrotophos	223.16	5371562

*Note*. http://www.ncbi.nlm.nih.gov/pccompound.

**Table 2:** Various genes present in the pMK-07 plasmid.

ORF	Sequence description	Gene length (bp)
AP007210.1_9	transposase	699
AP007210.1_11	group II intron reverse transcriptase maturase	1233
AP007210.1_14	SAM-dependent methyltransferase	1092
AP007210.1_18	chitin-binding protein	1275
AP007210.1_20	F0F1 ATP synthase subunit alpha	420
AP007210.1_21	acid-soluble spore C5	216
AP007210.1_22	chemotaxis protein	819
AP007210.1_23	Bacillolysin precursor	1671
AP007210.1_28	peptidase S8	762
AP007210.1_29	precorrin-3B C(17)-methyltransferase	468
AP007210.1_32	conserved hypothetical protein	144
AP007210.1_33	integrase	951
AP007210.1_37	nucleotidyltransferase	378
AP007210.1_38	cytotoxin	264
AP007210.1_40	DNA-binding protein	414
AP007210.1_41	nucleotidyltransferase	387
AP007210.1_43	thiamine biosynthesis	849
AP007210.1_50	DNA polymerase III subunit beta	1113
AP007210.1_56	membrane protein	285
AP007210.1_59	MULTISPECIES: membrane	264
AP007210.1_63	S1 RNA binding domain	984
AP007210.1_64	inosine-uridine preferring nucleoside hydrolase family	951
AP007210.1_65	reverse transcriptase	1647
AP007210.1_66	conjugation family	3501
AP007210.1_67	conserved hypothetical plasmid	387
AP007210.1_68	conserved hypothetical plasmid	1287
AP007210.1_69	Plasmid replication repX	1308
AP007210.1_70	conserved hypothetical protein	351
AP007210.1_72	IS605 family	1113
AP007210.1_78	IS605 family transposase	1335
AP007210.1_79	surface layer	1278
AP007210.1_81	integrase core domain	786
AP007210.1_82	DNA-binding	522
AP007210.1_84	Flp pilus assembly	861
AP007210.1_85	SAF domain family	852
AP007210.1_86	type II secretion system	1425
AP007210.1_87	membrane	933
AP007210.1_88	conserved hypothetical protein	867
AP007210.1_90	conserved domain	204
AP007210.1_91	conserved domain	207
AP007210.1_92	IS605 family transposase	1119
AP007210.1_95	membrane protein	624
AP007210.1_98	sortase	702
AP007210.1_99	LPXTG-motif cell wall anchor domain	528
AP007210.1_100	ATP-binding protein	189
AP007210.1_103	conserved hypothetical protein	252
AP007210.1_104	cell division	165
AP007210.1_106	recombination U	126
AP007210.1_113	transposase for insertion sequence element D	1275
AP007210.1_115	transposon resolvase	561
AP007210.1_116	S-layer homology domain ribonuclease	3396
AP007210.1_117	barnase inhibitor	276
AP007210.1_119	putative membrane protein	198
AP007210.1_124	MULTISPECIES: ATPase	924
AP007210.1_127	prgI family	351
AP007210.1_130	reverse transcriptase	1833
AP007210.1_132	Reticulocyte binding	3981
AP007210.1_133	M23 M37 family	2208
AP007210.1_139	CAAX amino protease	705
AP007210.1_141	penicillin-binding partial	414
AP007210.1_142	membrane protein	1146
AP007210.1_143	thiol reductase thioredoxin	483
AP007210.1_147	family transcriptional regulator	279
AP007210.1_148	transcriptional regulator	327
AP007210.1_149	integrase recombinase	1056
AP007210.1_150	Transposase (plasmid)	165
P007210.1_151	conserved domain	513
AP007210.1_152	transcriptional regulator	255
AP007210.1_153	conserved domain	156
AP007210.1_155	type VII secretion	1233
AP007210.1_156	SMI1 KNR4 family	450
AP007210.1_157	lumazine binding domain	381
AP007210.1_160	transcriptional family	300
AP007210.1_163	transposase, partial	630
AP007210.1_164	transposon resolvase	576
AP007210.1_165	family transcriptional regulator	846
AP007210.1_166	transposon resolvase	495
AP007210.1_168	N-acetylmuramoyl-L-alanine amidase	699
AP007210.1_169	phosphoglycerate mutase	570
AP007210.1_170	IS21 family	1254
AP007210.1_171	ATPase AAA	759
AP007210.1_172	Two-component response regulator	864
AP007210.1_173	glyoxalase family	354
AP007210.1_174	tn3 transposase DDE domain	3054
AP007210.1_175	site-specific recombinase	627
AP007210.1_177	XRE family transcriptional regulator	231
AP007210.1_178	alpha beta hydrolase	732
AP007210.1_180	cardiolipin synthetase	1494
AP007210.1_181	membrane yetF	549
AP007210.1_183	lipo	486
AP007210.1_184	stage V sporulation AC	477
AP007210.1_185	stage V sporulation AD	1017
AP007210.1_186	stage V sporulation AE	351
AP007210.1_187	NADH dehydrogenase	207
AP007210.1_189	ATP-dependent Clp protease proteolytic subunit	582
AP007210.1_190	resolvase	552
AP007210.1_191	spore germination C	1134
AP007210.1_192	spore germination	657
AP007210.1_193	spore germination family	1536
AP007210.1_194	phospholipase D competence helix-hairpin-helix domain	222
AP007210.1_195	transposase	1500
AP007210.1_196	transposase, partial	1695
AP007210.1_197	transposase for transposon	741
AP007210.1_198	transcriptional regulator	357
AP007210.1_199	S-(hydroxymethyl)glutathione dehydrogenase-like	1116
AP007210.1_200	S-glutathione hydrolase	834
AP007210.1_201	UDP-N-acetylmuramoylalanyl-D-glutamate--2,6-diaminopimelate ligase	273
AP007210.1_202	Glutamate--cysteine ligase	2268
AP007210.1_204	spore germination XA	696
AP007210.1_205	germination %2C Ger(x)C family	1155
AP007210.1_206	Spore germination	1161
AP007210.1_207	cell surface	15033
AP007210.1_209	Isochorismatase	531
AP007210.1_211	Two-component response regulator	681
AP007210.1_212	two-component sensor histidine kinase	1848
AP007210.1_214	conserved domain	1011
AP007210.1_215	inosine-uridine preferring nucleoside hydrolase family	333
AP007210.1_217	cell surface	3711
AP007210.1_218	cell surface	6834
AP007210.1_219	RNA-binding Hfq	186
AP007210.1_220	family transcriptional regulator	294
AP007210.1_221	2-oxoglutarate dehydrogenase	429
AP007210.1_225	DNA topoisomerase I	2664

**Table 3:** Docking score of *α*-*β* hydrolase with various ligands.

Ligands/Pesticide	Gscore Kcal/mol	Number of Hydrogen Bonds	Amino Acid Interacting with Ligand
Chloramphenicol	−3.793	4	Gly11, Ile12, Ser 77
Streptomycin	−2.865	3	Gly13, Ile12
Cefotaxime	−5.885	2	Asn18, Ser 77
Ampicillin	−4.316	2	Asn18, Ser 77
Tetracycline	−3.972	1	Ile12
Monocrotophos	−4.464	2	Ile12, Ser 77
